# Supplementary material for: Wide Application of Minimally Processed Saliva on Multiple RT-qPCR Kits for SARS-CoV-2 Detection in Indonesia
Source: Front Cell Infect Microbiol. 2021 Aug 18;11:691538. doi: 10.3389/fcimb.2021.691538 (PMC8416441; doi:10.3389/fcimb.2021.691538)
Supplement: Supplementary file 1 [file DataSheet_1.docx]

**SUPPLEMENTARY INFORMATION**

**Supplementary 1. Optimization of RNA-Extraction-free treatment of saliva specimens.**

| Treatment | Sample type | Extraction |
| --- | --- | --- |
| 1 | NPOP | Qiagen |
| 2 | Saliva | Qiagen |
| 3 | Saliva | 1.Transfer 100μl  2. Add 10ul Proteinase K  3. Incubate sample at 37°C for 30 mins  4. Heat inactivation at 95°C for 10 mins |
| 4 | Saliva | 1. Transfer 100μl  2. Add 10ul Proteinase K  3. Incubate sample at 37°C for 15 mins  4. Heat inactivation at 95°C for 10 mins |
| 5 | Saliva | 1. Transfer 100μl  2. Incubate sample at 37°C for 30 mins  3. Heat inactivation at 95°C for 10 mins |
| 6 | Saliva | 1. Transfer 100μl  2. Heat inactivation at 95°C for 10 mins |
| 7 | Saliva | 1. Transfer 100μl  2. Heat inactivation at 95°C for 10 mins  3. Centrifuge at max speed (13200 rpm) for 2 mins |
| 8 | Saliva | 1. Transfer 100μl  2. Heat inactivation at 95°C for 10 mins  3. Centrifuge at max speed (13200 rpm) for 5 mins |

**Supplementary 2. Specifications and characteristics of the commercial RT-qPCR kits, as derived from their respective manufacturer's instructions**

| Kit name | Abbreviation* | Target Genes – Reporter Dye | Internal Control – Reporter Dye | LOD (copies/ml) | Reaction Volume (µl) | Template Volume (µl) | No. of Cycles | Cycle Cut–off |
| --- | --- | --- | --- | --- | --- | --- | --- | --- |
| Detection Kit for 2019 Novel Coronavirus (2019-nCoV) | Da An Gene | N gene – FAM  ORF1ab – VIC | RNase P – Cy5 | 500 | 25 | 5 | 45 | 40 |
| Maccura SARS-CoV-2 Fluorescent PCR kit | Maccura | ORF1ab – FAM  E gene – ROX  N gene – Cy5 | MS2 based pseudo virus – VIC/HEX | 1000 | 20 | 20 | 40 | 38 |
| Fosun COVID-19 RT-PCR Detection Kit | Fosun | ORF1ab – FAM  E gene – ROX  N gene – JOE | Lentivirus – Cy5 | 300 | 20 | 10 | 40 | 36 |
| Novel Coronavirus (COVID-19) Nucleic Acid Detection Kit (PCR-fluorescent probe) | Ardent | ORF1ab (RdRp) – FAM  N gene – VIC | RNase P – Cy5 | 400 | 15 | 5 | 45 | 40 |
| Standard M nCoV Real-Time Detection kit | SD Biosensor | ORF1ab (RdRP) – FAM  E gene-VIC/HEX | Internal control A (Pseudovirus) – Cy5 | 250 | 20 | 10 | 40 | 36 |
| Real-Q 2019 nCoV Detection Kit | Biosewoom | RdRP gene – FAM  E gene – HEX/VIC | HRP – Cy5 | 3125 | 20 | 5 | 40 | 38 |
| MiRXES Fortitude kit 2.1 | Fortitude | ORF1ab region 1 –FAM  ORF1ab region 2 – HEX | Synthetic DNA oligo – Cy5 | 200 | 20 | 5 | 42 | 40 |
| 3S SARS-CoV-2 RT-PCR Kit | 3S | N gene – FAM  ORF1ab – HEX | ꞵ-actin – VIC/HEX | 300 | 15 | 10 | 45 | 40 |
| 2019-Novel Coronavirus (2019-nCoV) Triplex RT-qPCR Detection Kit | Vazyme | ORF1ab – FAM  N gene – ROX/Texas Red | RNase P – Cy5 | 200 | 30 | 20 | 45 | 38 |
| * Based on kit name |  |  |  |  |  |  |  |  |

**Supplementary 3. SARS-CoV-2 detection from RNA extracted from NPOP *versus* saliva specimens.**

| Extracted saliva, n | NPOP | | |  |
| --- | --- | --- | --- | --- |
|  | Positive | Negative | Invalid | Total |
| Positive | 60 | 0 | 2 | 62 |
| Negative | 11 | 32 | 2 | 45 |
| Invalid | 7 | 1 | 1 | 9 |
| Total | 78 | 33 | 5 | 116 |

**Supplementary 4. Comparison of eight different sample treatments prior to SAR-CoV-2 detection via PCR for negative saliva specimens.** Bar graphs represent means ± SEM for three SARS-CoV-2 negative specimens tested for each sample treatment illustrated in **Figure 1A**.


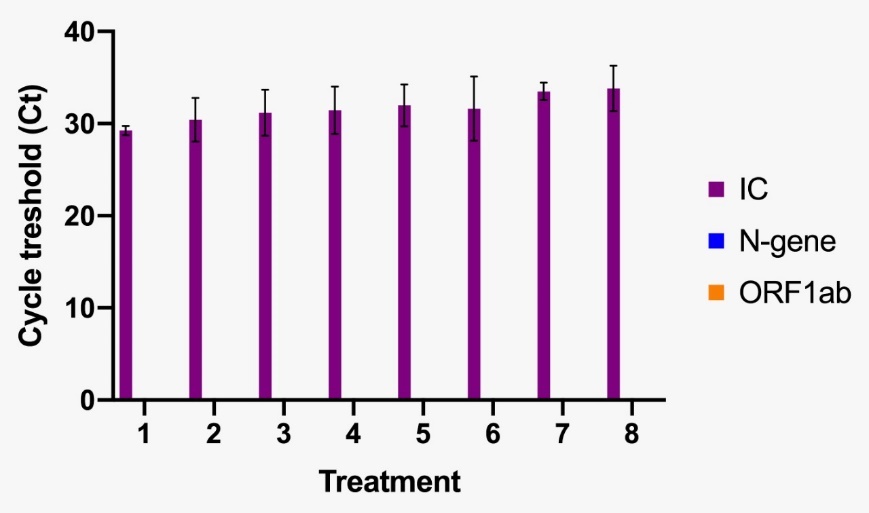


**Supplementary 5. Detection of SARS-CoV-2 target genes N and ORF1ab plotted as the Ct value obtained for monitored sample.** Data points collected in Day 1 are displayed as white circles with black outlines. Blue points refer to samples stored at cold while green at room temperature. Straight lines are drawn to monitor changes for samples starting with the highest and lowest Ct value in Day 1.

**
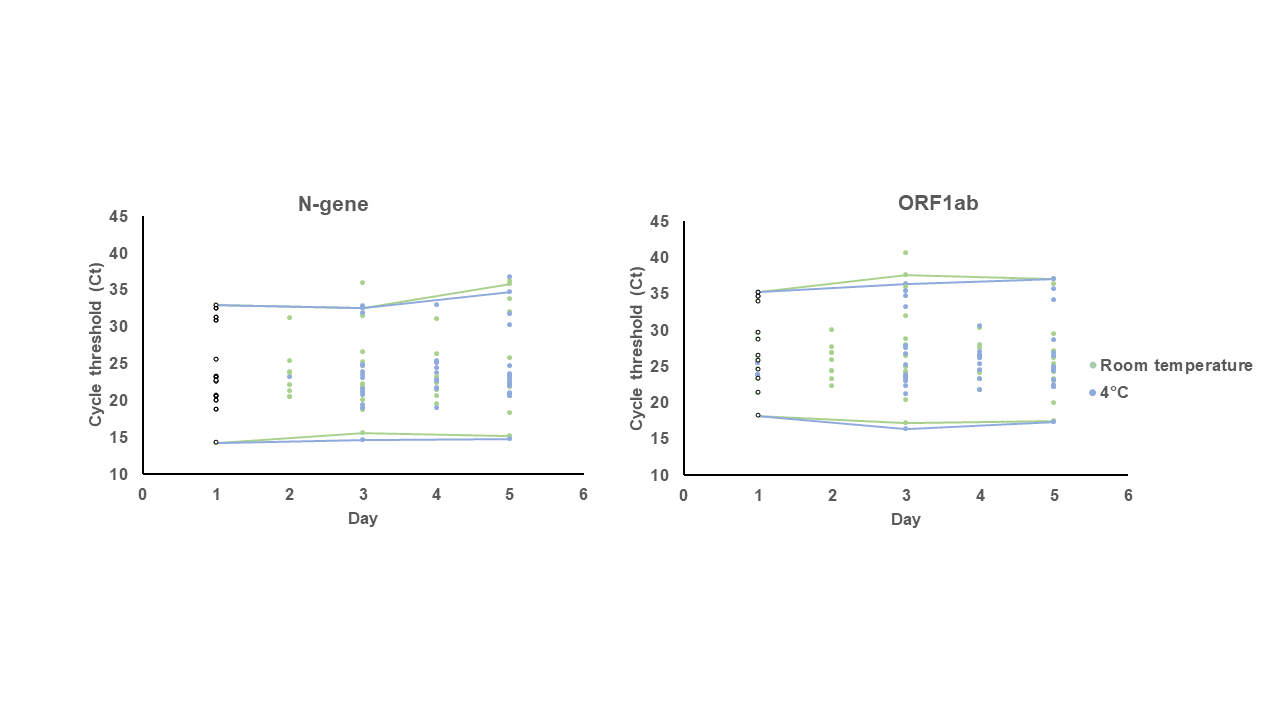
**

**Supplementary 6. Validation as RNA-extraction-free treatment of saliva.**

| Treated saliva, n | NPOP | | |  |
| --- | --- | --- | --- | --- |
|  | Positive | Negative | Invalid | Total |
| Positive | 57 | 0 | 1 | 58 |
| Negative | 15 | 38 | 3 | 56 |
| Invalid | 9 | 1 | 1 | 11 |
| Total | 81 | 39 | 5 | 125 |

**Supplementary 7. Comparison of conical tubes and sampling device for RNA-extraction-free treatment of saliva.**

| Treated saliva, n (conical tubes^1^) | NPOP | |  |  | Treated saliva, n (sampling device^2^) | NPOP | |  |
| --- | --- | --- | --- | --- | --- | --- | --- | --- |
|  | Positive | Negative | Total |  |  | Positive | Negative | Total |
| Positive | 23 | 0 | 23 |  | Positive | 34 | 0 | 34 |
| Negative | 9 | 12 | 21 |  | Negative | 6 | 26 | 32 |
| Total | 32 | 12 | 44 |  | Total | 40 | 26 | 66 |
|  |  |  |  |  |  |  |  |  |
| Agreements | % | 95% CI | |  | Agreements | % | 95% CI | |
| Overall* | 79.55 | 65.50-88.85 | |  | Overall^#^ | 90.91 | 81.55-95.68 | |
| Positive** | 71.88 | 54.63-84.44 | |  | Positive^##^ | 85.00 | 70.93-92.94 | |
| Negative*** | 100.00 | 75.76-100.00 | |  | Negative^###^ | 100.00 | 87.13-100.00 | |
| ^1^ Volume collected 2-5 mL  *Overall agreement = ((23+12)/44) x 100  **Positive agreement = (29/32) x 100  ***Negative agreement = (12/12) x 100 | |  | |  | ^2^ Volume collected 0.5-1.0 mL  ^#^Overall agreement = ((34+26)/66) x 100  ^##^Positive agreement = (34/40) x 100  ^###^Negative agreement = (26/26) x 100 | |  | |

**Supplementary 8. Performance of commercial kits using treated saliva as template for RT-qPCR.**

| Commercial kit | Sample size, n | True positive, n | False positive, n | True negative, n | False negative, n | Invalid samples, n | Invalid rate, % | Overall agreement, % | Estimated sensitivity, % |
| --- | --- | --- | --- | --- | --- | --- | --- | --- | --- |
| Maccura | 11 | 11 | 0 | 0 | 0 | 0 | 0.00 | 100.00 | 100.00 |
| Fosun | 12 | 6 | 0 | 5 | 0 | 1 | 8.33 | 100.00 | 100.00 |
| Ardent | 11 | 10 | 0 | 0 | 1 | 0 | 0.00 | 90.91 | 90.91 |
| SD Biosensor | 13 | 8 | 0 | 3 | 1 | 1 | 7.69 | 91.67 | 88.89 |
| Biosewoom | 11 | 8 | 0 | 0 | 2 | 1 | 9.09 | 80.00 | 80.00 |
| Fortitude | 11 | 7 | 0 | 0 | 3 | 1 | 9.09 | 70.00 | 70.00 |
| 3S | 10 | 2 | 6 | 2 | 0 | 0 | 0.00 | 40.00 | 100.00 |
| Vazyme | 10 | 2 | 4 | 0 | 0 | 4 | 40.00 | 33.33 | 100.00 |

**Supplementary 9. Implementation of sampling device for collection of saliva specimen.**

| Treated saliva collected with sampling device, n | NPOP | | |  |
| --- | --- | --- | --- | --- |
|  | Positive | Negative | Invalid | Total |
| Positive | 45 | 0 | 0 | 45 |
| Negative | 8 | 240 | 2 | 250 |
| Invalid | 10 | 1 | 0 | 11 |
| Total | 63 | 241 | 2 | 306 |

**Supplementary 10. Calculation of invalid samples in the different specimens and treatment conditions.**

|  | Extracted NPOP | Extracted saliva | Treated saliva | Treated saliva collected with sampling device |
| --- | --- | --- | --- | --- |
| valid | 535 | 107 | 114 | 295 |
| invalid | 29 | 9 | 11 | 11 |
| total, n | 564 | 116 | 125 | 306 |
| % invalid | 5.14 | 7.76 | 8.80 | 3.59 |
